# Redox/methylation mediated abnormal DNA methylation as regulators of ambient fine particulate matter-induced neurodevelopment related impairment in human neuronal cells

**DOI:** 10.1038/srep33402

**Published:** 2016-09-14

**Authors:** Hongying Wei, Fan Liang, Ge Meng, Zhiqing Nie, Ren Zhou, Wei Cheng, Xiaomeng Wu, Yan Feng, Yan Wang

**Affiliations:** 1 Shanghai Jiao Tong University School of Public Health; Hongqiao International Institute of Medicine, Shanghai Tongren Hospital, Shanghai 200025, China; 2 Shanghai Jiao Tong University School of Environmental Science and Engineering, Shanghai, 200240, China; 3 Shanghai Ninth People’s Hospital, Shanghai Jiao Tong University School of Medicine, Shanghai, 200011, China

## Abstract

Fine particulate matter (PM_2.5_) has been implicated as a risk factor for neurodevelopmental disorders including autism in children. However, the underlying biological mechanism remains unclear. DNA methylation is suggested to be a fundamental mechanism for the neuronal responses to environmental cues. We prepared whole particle of PM_2.5_ (PM_2.5_), water-soluble extracts (Pw), organic extracts (Po) and carbon core component (Pc) and characterized their chemical constitutes. We found that PM_2.5_ induced significant redox imbalance, decreased the levels of intercellular methyl donor S-adenosylmethionine and caused global DNA hypomethylation. Furthermore, PM_2.5_ exposure triggered gene-specific promoter DNA hypo- or hypermethylation and abnormal mRNA expression of autism candidate genes. PM_2.5_-induced DNA hypermethylation in promoter regions of synapse related genes were associated with the decreases in their mRNA and protein expression. The inhibiting effects of antioxidative reagents, a methylation-supporting agent and a DNA methyltransferase inhibitor demonstrated the involvement of redox/methylation mechanism in PM_2.5_-induced abnormal DNA methylation patterns and synaptic protein expression. The biological effects above generally followed a sequence of PM_2.5_ ≥ Pwo > Po > Pw > Pc. Our results implicated a novel epigenetic mechanism for the neurodevelopmental toxicity of particulate air pollution, and that eliminating the chemical components could mitigate the neurotoxicity of PM_2.5_.

Air pollution, especially ambient particulate matter, has been reported to be associated with neuropathology and central nervous system diseases, including stroke^1^, neurodegenerative diseases^2^, and autism spectrum disorders (ASD)^3^. Residential proximity to freeways, gestational and early life exposure to air pollution and perinatal exposure to fine particulate matter (PM_2.5_) may increase the risk for autism in children^4^,^5^,^6^. Genetic and environmental factors have been implicated in the development of neurodevelopmental pathology, but the molecular mechanisms underlying their interaction are not clear. Epigenetic modifications have been suggested as the molecular mechanisms for air pollution-induced neurodevelopmental disorders^7^.

Human data has provided strong biological plausibility for the link between abnormal DNA methylation, one of the epigenetic modifications, and PM_2.5_-related health effects. Previous studies have shown the associations between PM_2.5_ exposure and decreased repeated-element methylation or placental global DNA hypomethylation^8^,^9^. In addition to global DNA methylation, acute and chronic exposure to PM_2.5_ generated from welding activities have been correlated with increased methylation in the promoter region of the inducible nitric oxide synthase gene^10^. Until now, evidence has been provided for PM-induced epigenetic alterations in human blood^8^, human circulating mononuclear cells^11^, animal lung tissue^12^, and murine macrophage cell lines^13^. However, the association between developmental PM_2.5_-induced neurotoxicity and DNA methylation as well as correlative gene expression remains to be identified, and the underlying molecular mechanisms are also largely unknown.

A large number of studies have shown that oxidative stress is one of the most important mechanisms for the adverse health effects of ambient particulate matter^14^. Meanwhile, oxidative stress status, an imbalance in glutathione redox metabolism and impairments in genomewide DNA methylation as well as gene-specific DNA methylation have been reported in children with ASD^15^,^16^. Thus, we speculated that the oxidative stress was possibly associated with neurodevelopmental dysfunction induced by ambient particulate matter. In addition, DNA methylation involves the addition of methyl groups to cytosine residues in CpG dinucleotides to form 5-methylcytosine (5mC). Methyl groups from S-adenosylmethionine (SAM) are required for DNA methylation. However, the enhanced require for glutathione (GSH) synthesis resulted from oxidative stress needs more homocysteine as substrates, and consequently reduces the availability of homocysteine for use in the SAM synthesis and thereby perturbs DNA methylation^17^,^18^. Based on the metabolic relationship between oxidative stress and the methylation groups, we further proposed that the oxidative stress-induced methylation abnormality (redox/methylation mechanism) might be involved in PM_2.5_-induced neurodevelopmental disorders.

In the current study, we aimed to investigate whether exposure of human neuronal cells to PM_2.5_ could induce abnormal DNA methylation patterns through redox/methylation mechanism. The investigation of abnormal DNA methylation patterns included global DNA methylation and gene-specific DNA methylation of autism candidate genes. In addition, we analyzed the effects of different types of PM_2.5_ extracts to evaluate the contribution of different chemical compositions to the toxicity of PM_2.5_.

## Results

### The chemical constituents and characteristics of PM_2.5_ and its extracts

**Figure 1:**
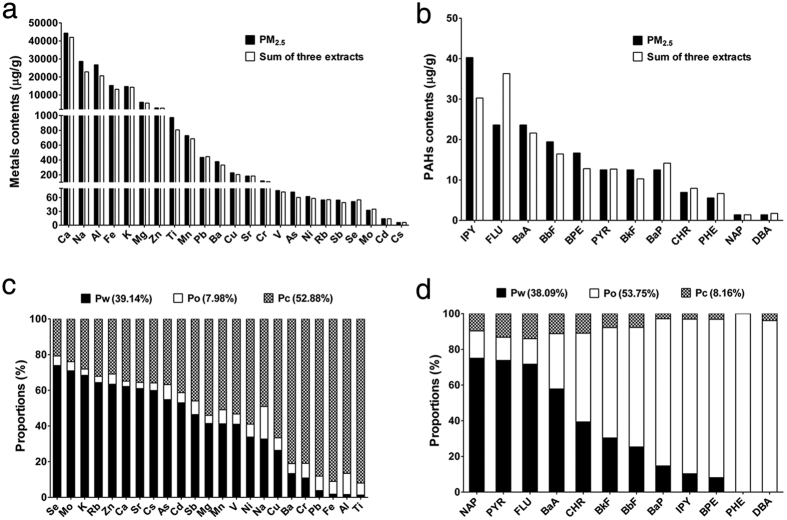
The chemical constituents of PM_2.5_ and its extracts. Comparison of the 23 types of metals (**a**) and 16 types of PAHs (**b**) between PM_2.5_ (by determination) and the sum of three extracts (Pw, Po and Pc). The proportions of metals (**c**) and PAHs (**d**) in different extracts (Pw, Po and Pc). Pw: the water-soluble extracts of PM_2.5_. Po: the organic extracts of PM_2.5_. Pc: the carbon core component of PM_2.5_.

PM_2.5_ sampling filters were used to prepare whole particles of PM_2.5_ (PM_2.5_), water-soluble extracts (Pw), organic extracts (Po) and carbon core component (Pc), successively. The contents of the various chemical components of PM_2.5_ and its extracts are presented in Supplementary Table S1 (Supplementary material 2). The contents of the metals and polycyclic aromatic hydrocarbons (PAHs) generally showed similar results between PM_2.5_ and the sum of the three extracts (Pw, Po and Pc), which indicates the good quality of sample preparation and chemical analysis (Fig. 1a,b). According to the results of chemical analysis, we found that the distribution of metals and PAHs in different extracts showed obviously different features. As shown in Fig. 1c, Pw accounted for 39.14% of the total metals, with Se, Mo, K, Rb, Zn, Ca, Sr, Cs, As and Cd contributing the majority. Pc accounted for 52.88% of the total metals, mainly including Ti, Al, Fe, Pb, Cr, Ba, Cu, Na, Ni, V and Mn (Fig. 1c). Po contained a small amount of metals, accounting for 7.98% of the total metals (Fig. 1c). Po contained more than half (53.75%) of the total PAHs with DBA, PHE, BPE, IPY, BaP, BbF, BkF and CHR as the main compositions (Fig. 1d). Pw also accounted for 38.09% of the total PAHs, especially including NAP, PYR, FLU and BaA (Fig. 1d). Pc contained a minimal amount (8.16%) of the total PAHs (Fig. 1d). The concentrations of ANY, ANA, ANT and FLT were below their detection limits in all samples.

### PM_2.5_ and its extracts induced oxidative stress and disturbed redox balance

**Figure 2:**
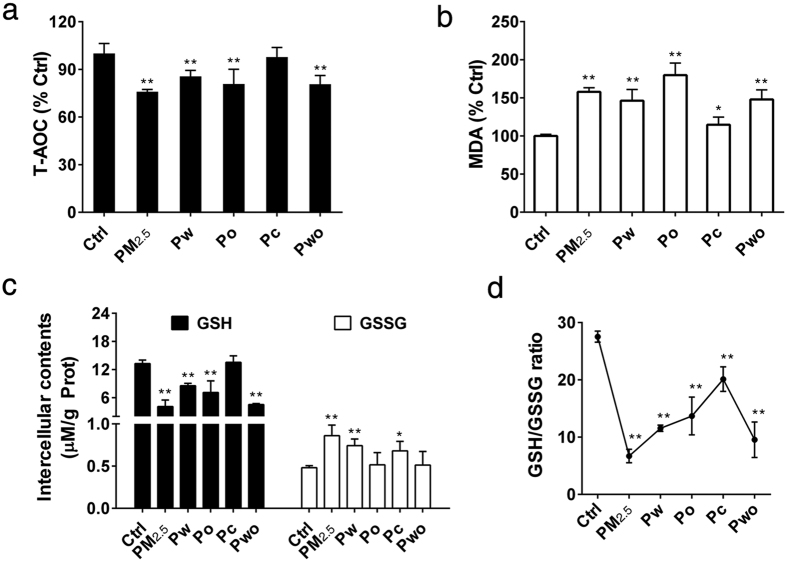
PM_2.5_ and its extracts induced oxidative stress and disturbed redox balance (SH-SY5Y cells; 72 h treatment). The intracellular T-AOC ((**a**), n = 6), MDA ((**b**), n = 4), GSH ((**c**), n = 4), GSSG ((**c**), n = 4) and GSH/GSSG ((**d**), n = 4). *P < 0.05, **P < 0.01, effects *versus* control (by one-way ANOVA with LSD *post hoc* test).

Firstly, the cytotoxicity of PM_2.5_ and its extracts were evaluated. We found that PM_2.5_ and its extracts caused significant cytotoxicity at high concentrations (Supplementary material 1: Fig. S1). According to the results, we chose 80 μg/mL of PM_2.5_, 40 μg/mL of Pw (50%), 12 μg/mL of Po (15%), 28 μg/mL of Pc (35%) and 40 μg/mL of Pw combined with 12 μg/mL of Po (Pwo) in the subsequent experiments. The cell viability was more than 85% under these conditions, and there was no obvious cell toxicity in corresponding negative control of ddH_2_O (2%) and dimethyl sulfoxide (DMSO; 0.1%) (Supplementary material 1: Fig. S2). We then exposed SH-SY5Y cells to PM_2.5_ and its extracts with these concentrations for 72 h. The oxidative stress effects were evaluated by using an indicator of intracellular total antioxidant capacity (T-AOC), the lipid peroxidation malonadialdehyde (MDA) and the ratio of reduced glutathione to oxidized glutathione (GSH/GSSG). As shown in Fig. 2, significantly decreases in intercellular T-AOC (Fig. 2a) and increases in MDA contents (Fig. 2b) were observed after PM_2.5_, Pw, Po and Pwo exposure. Pc induced the weakest effects on the decreases in T-AOC and the increases in MDA (Fig. 2a,b). The results of GSH and GSSG showed the disturbance in cellular redox balance, with obvious decreases in GSH (Fig. 2c), increases in GSSG (Fig. 2c) and decreases in GSH/GSSG ratio (Fig. 2d). The lowest ratio (6.71) was observed in the PM_2.5_-exposed group, followed by Pwo (9.55), Pw (11.54) and Po (13.69) (Fig. 2d). Pc also caused the weakest effects on redox imbalance and the ratio was 20.13 (Fig. 2d). Likewise, positive control hydrogen peroxide (H_2_O_2_) substantially decreased the contents of GSH and the ratio of GSH/GSSG, while the negative control ddH_2_O and DMSO did not cause a significant oxidative stress effects (Supplementary material 1: Fig. S3). A decrease in GSH/GSSG is suggestive of redox imbalance and diminished antioxidant defenses. These results indicated that PM_2.5_ and its extracts induced significant oxidative stress and disturbed the redox balance in SH-SY5Y cells.

### PM_2.5_ and its extracts impaired methylation capacity and induced global DNA hypomethylation

**Figure 3:**
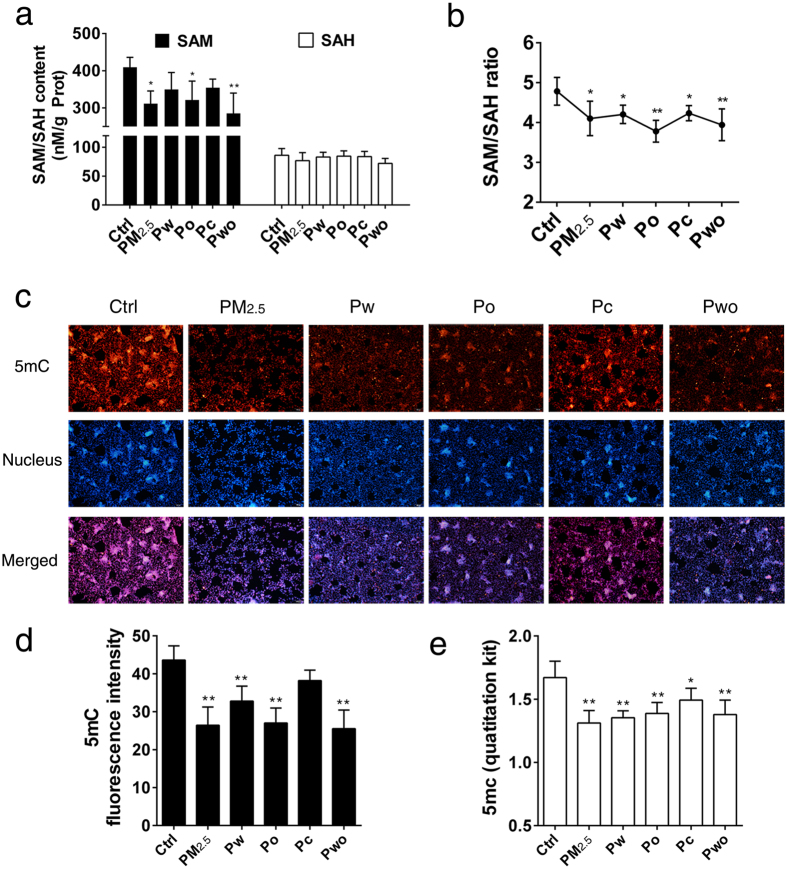
PM_2.5_ and its extracts impaired methylation capacity and induced global DNA hypomethylation (SH-SY5Y cells; 72 h treatment). The intracellular SAM ((**a**), n = 3), SAH ((**a**), n = 3) and SAM/SAH ((**b**), n = 3). (**c**) The representative images of 5mC immunofluorescence (200×). Red: Cy3-5mC. Blue: Hoechst 33258-cell nuclei. (**d**) The quantification analysis of 5mC fluorescence intensity (n = 3). (**e**) The 5mC levels detected by quantitation kit (n = 3). *P < 0.05, **P < 0.01, effects *versus* control (by one-way ANOVA with LSD *post hoc* test).

Oxidative stress and the redox imbalance have been related to the impairment of the SAM synthesis^17^. The use of the ratio of SAM/SAH (S-adenosyl homocysteine) has been known as a reporter of the methylation capacity of the cell^17^,^19^. As shown in Fig. 3, PM_2.5_ and its extracts induced obvious decreases in SAM concentration (Fig. 3a), and thus resulted in a reduced SAM/SAH ratio (Fig. 3b). A lower SAM/SAH ratio is considered as an indication of reduced methylation potential^17^,^20^. Next, we examined 5mC contents to evaluate the status of global genome methylation. The fluorescence intensity of 5mC in the nuclei was significantly decreased after treatment with PM_2.5_, Pw, Po or Pwo (Fig. 3c). The 5mC fluorescence intensity (Fig. 3d) showed a trend similar to the change in 5mC quantitative analysis by kit (Fig. 3e). As expected, treatment of the cells with H_2_O_2_ led to obvious decreases in SAM concentration, SAM/SAH ratio and global 5mC level, and ddH_2_O as well as DMSO had no effects on methylation capacity (Supplementary material 1: Fig. S4). This work showed that PM_2.5_ and its extracts impaired methylation capacity and induced global DNA hypomethylation.

### PM_2.5_ and its extracts interfered with gene-specific DNA methylation and mRNA expression of autism candidate genes

**Figure 4:**
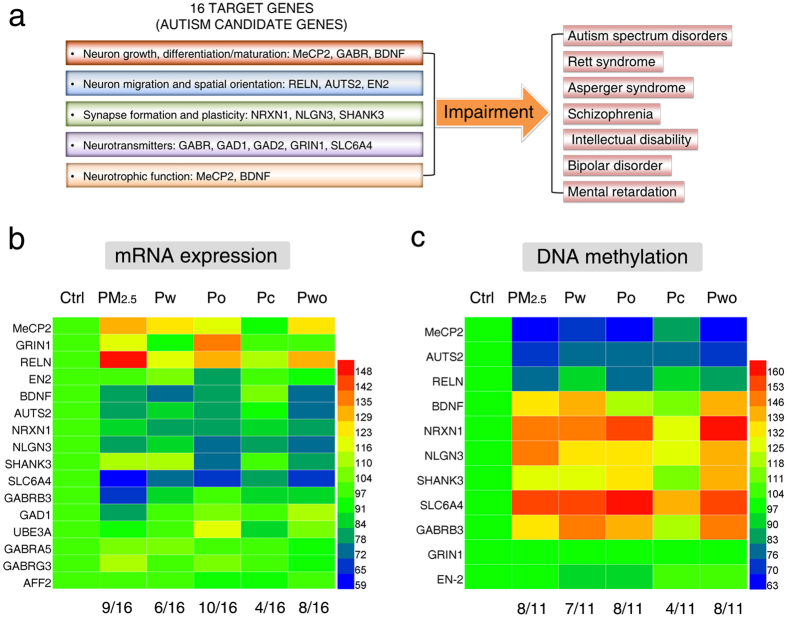
PM_2.5_ and its extracts interfered with gene-specific DNA methylation and the expression of autism candidate genes (SH-SY5Y cells; 72 h treatment). (**a**) The relationship between 16 target genes and neurodevelopment disorders. The heatmap of mRNA expression of 16 target genes (**b**) and promoter DNA methylation of 11 target genes (**c**) (n = 3). The numbers below the heatmap indicate the percent of the results with statistical significance (*P* < 0.05) compared with control (by one-way ANOVA with LSD *post hoc* test).

Oxidative stress and abnormal global DNA methylation possibly influences the genomic stability and gene-specific DNA methylation^19^. Thus, we evaluated the mRNA expression and promoter DNA methylation of autism candidate genes which are associated with neurodevelopmental and synaptic functions. The relationship between 16 target genes and neurodevelopment disorders is shown in Fig. 4a and Supplementary Table S2 (Supplementary material 2). After treatments, none of the treatments caused significant changes in mRNA expression of GAD1, UBE3B, GABRA5, GABRG3 and AFF2 (Fig. 4b). The mRNA expression of MeCP2, GRIN1 and RELN were upregulated by PM_2.5_ and its extracts, and the mRNA expression of eight other genes (EN2, BDNF, AUTS2, NRXN1, NLGN3, SHANK3, SLC6A4 and GABRB3) were downregulated by treatment with PM_2.5_ and its extracts (Fig. 4b). Overall, Po and PM_2.5_ significantly impacted 10/16 and 9/16 genes, respectively. The other extracts significantly impacted several target genes (Pwo: 8/16; Pw: 6/16; Pc: 4/16) (Fig. 4b). For each particular gene, PM_2.5_, Pwo and Po generally induced stronger effects than Pw and Pc (Fig. 4b). Next, 11 genes with the alterations in mRNA expression were analyzed for DNA methylation in the promoter regions using Methylated DNA immunoprecipitation (MeDIP). DNA methylation in the promoters of MeCP2, AUTS2 and RELN was downregulated, and DNA methylation in the promoters of six other genes (BDNF, NRXN1, NLGN3, SHANK3, SLC6A4 and GABRB3) was upregulated (Fig. 4c). PM_2.5_, Po and Pwo impacted 8/11 genes, while Pw and Pc impacted 7/11 and 4/11 genes, respectively (Fig. 4c). Pw and Pc induced weaker effects than PM_2.5_, Po and Pwo for a specific gene (Fig. 4c). The data of mRNA expression and DNA methylation are presented in Supplementary Tables S3 and S4, respectively (Supplementary material 2). Therefore, PM_2.5_ and its extracts interfered with gene-specific DNA methylation and mRNA expression of autism candidate genes, with a rank order of PM_2.5_ ≥ Pwo ≥ Po > Pw > Pc.

### PM_2.5_ and its extracts upregulated promoter DNA methylation and reduced the expression of synapse related genes

**Figure 5:**
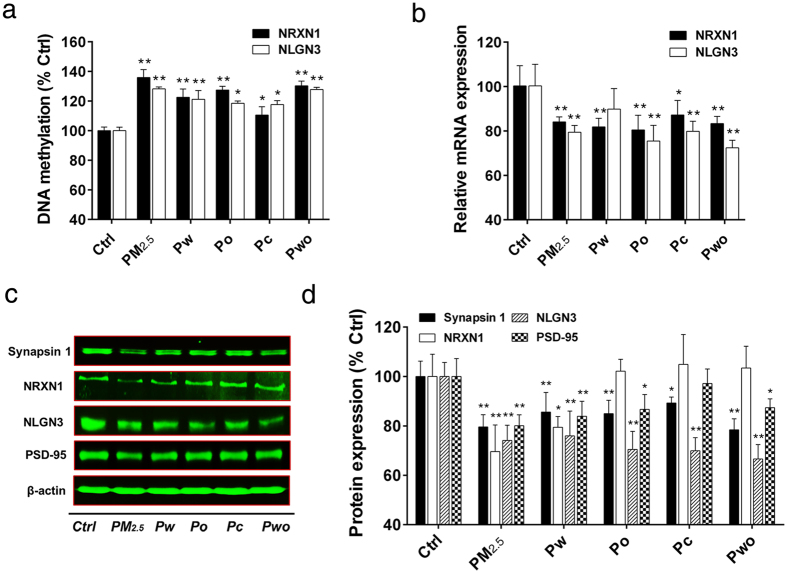
PM_2.5_ and its extracts upregulated promoter DNA methylation and reduced the expression of synapse related genes (SH-SY5Y cells; 72 h treatment). (**a**) DNA methylation in promoter regions of NRXN1 and NLGN3 (n = 3). (**b**) Relative mRNA expression of NRXN1 and NLGN3 (n = 3). The representative image (**c**) and quantitation results ((**d**), n = 3) of protein expression of synaptic proteins (synapsin 1, NRXN1, NLGN3 and PSD-95). *P < 0.05, **P < 0.01, effects *versus* control (by one-way ANOVA with LSD *post hoc* test).

The brain processes information by transmitting signals at synapses to form vast networks of communicating cells. Synapse dysfunction is one of the important mechanisms for neurodevelopmental dysfunctions. We had observed that synaptic adhesion molecules NRXN1 and NLGN3 showed promoter DNA hypermethylation and decreased mRNA expression (Fig. 4). We further confirmed the promoter DNA methylation and protein expression of NRXN1 and NLGN3 by pyrosequencing and western blot, respectively. Pyrosequencing indicated that DNA methylation in the promoter regions of NRXN1 and NLGN3 was significantly upregulated, with the PM_2.5_ and Pwo treatments showing the strongest effects (130% to 150% compared with control), whereas Pc induced the weakest effects (105% to 110% compared with control) (Fig. 5a). PM_2.5_ and its extracts also induced significant decreases in mRNA expression of NRXN1 and NLGN3 (Fig. 5b). Like PM_2.5_ treatment, the positive control H_2_O_2_ caused significant increases in promoter methylation and decreases in mRNA expression of NRXN1 and NLGN3 (Supplementary material 1: Fig. S5). The negative results of ddH_2_O and DMSO treatments further demonstrated the effects of PM_2.5_ and its extracts (Supplementary material 1: Fig. S5). Furthermore, the protein expressions of NRXN1 and NLGN3 as well as other synapse markers (synapsin 1 and PSD-95) were measured. The levels of synapsin 1, NLGN3 and PSD-95 were significantly decreased to about 70% by PM_2.5_ and its extracts, with Pc treatment showing the weakest effects (Fig. 5c,d). PM_2.5_ and Pw significantly decreased the protein expression of NRXN1 (70% to 80% compared with control) (Fig. 5c,d). Together, PM_2.5_ and its extracts upregulated promoter DNA methylation and reduced the expression of synapse related genes.

### Antioxidative reagents blocked PM_2.5_-induced oxidative stress, abnormal DNA methylation and the expression of synapse related genes

**Figure 6:**
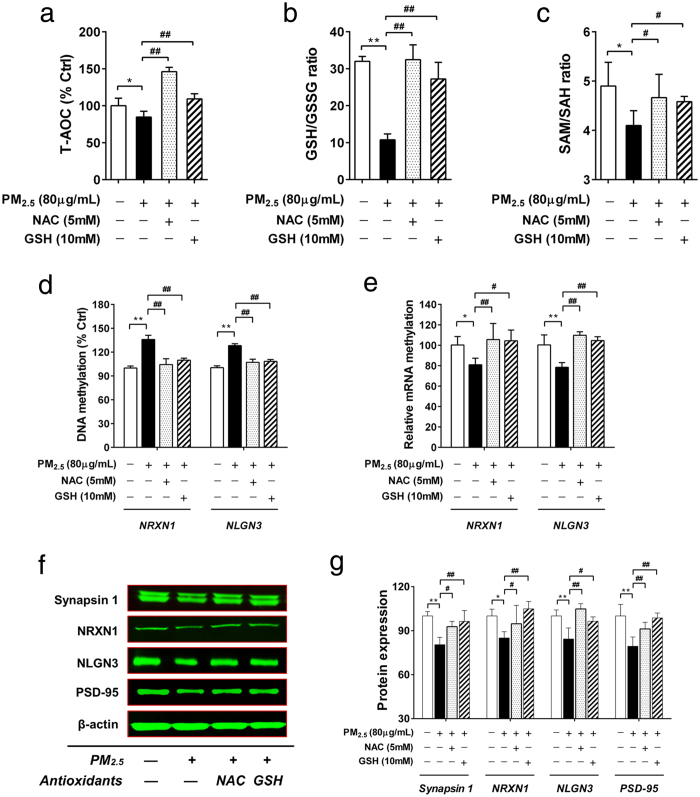
The blocking effects of antioxidative reagents (SH-SY5Y cells; 72 h treatment). The effects of NAC and GSH on PM_2.5_-induced changes in T-AOC ((**a**), n = 6), GSH/GSSG ((**b**), n = 4) and SAM/SAH ((**c**), n = 3). The effects of NAC and GSH on PM_2.5_-induced changes in DNA methylation (**d**) and mRNA expression (**e**) of NRXN1 and NLGN3 (n = 3). (**f,g**) The effects of NAC, GSH and SAM on PM_2.5_-induced changes in protein expression of synapsin 1, NRXN1, NLGN3 and PSD-95. (**f**) The representative images of western blot. (**g**) The quantitation results of protein expression (n = 3). *P < 0.05, **P < 0.01, effects *versus* control; ^#^*P* < 0.05, ^##^*P* < 0.01, effects *versus* PM_2.5_-treated group (by student’s *t* test).

To reveal the involvement of redox/methylation mechanisms in PM_2.5_-induced abnormal changes described above, we firstly evaluated the effects of antioxidative reagents. As shown in Fig. 6a,b, N-acetyl-L-cysteine (NAC) significantly prevented PM_2.5_-induced decreases in T-AOC and restored the redox balance (GSH/GSSG). Supplementation with GSH produced similar effects to those of NAC on T-AOC and redox balance (Fig. 6a,b). Furthermore, NAC and GSH reversed PM_2.5_-induced abnormal changes in methylation capacity, as indicated by significant increases in SAM/SAH ratio (Fig. 6c). Meanwhile, NAC and GSH also restored the redox balance and methylation capacity in H_2_O_2_-treated cells (Supplementary material 1: Fig. S6a,b). The results of the 5mC immunofluorescence analysis further confirmed the improvement of genome-wide DNA hypomethylation (Supplementary material 1: Fig. S7).

Furthermore, the effects of antioxidative reagents on PM_2.5_-induced promoter DNA hypermethylation and decreased expression of synapse related genes were evaluated. As shown in Fig. 6d, NAC and GSH significantly prevented promoter DNA hypermethylation of NRXN1 and NLGN3 induced by PM_2.5_. The decreases in mRNA expression of NRXN1 and NLGN3 were also reversed by NAC and GSH (Fig. 6e). PM_2.5_-induced decreases in protein expression of synapse related genes were restored by NAC and GSH (Fig. 6f,g). Together with the blocking effects of NAC and GSH in H_2_O_2_-induced abnormal changes in promoter methylation and mRNA expression of NRXN1 and NLGN3 (Supplementary material 1: Fig. S6c,d), we demonstrated the involvement of redox/methylation mechanisms in PM_2.5_-induced DNA hypermethylation and the decreases in gene expression of synapse related genes.

### The methylation supporting agent prevented PM_2.5_-induced redox imbalance, abnormal DNA methylation and the expression of synapse related genes

**Figure 7:**
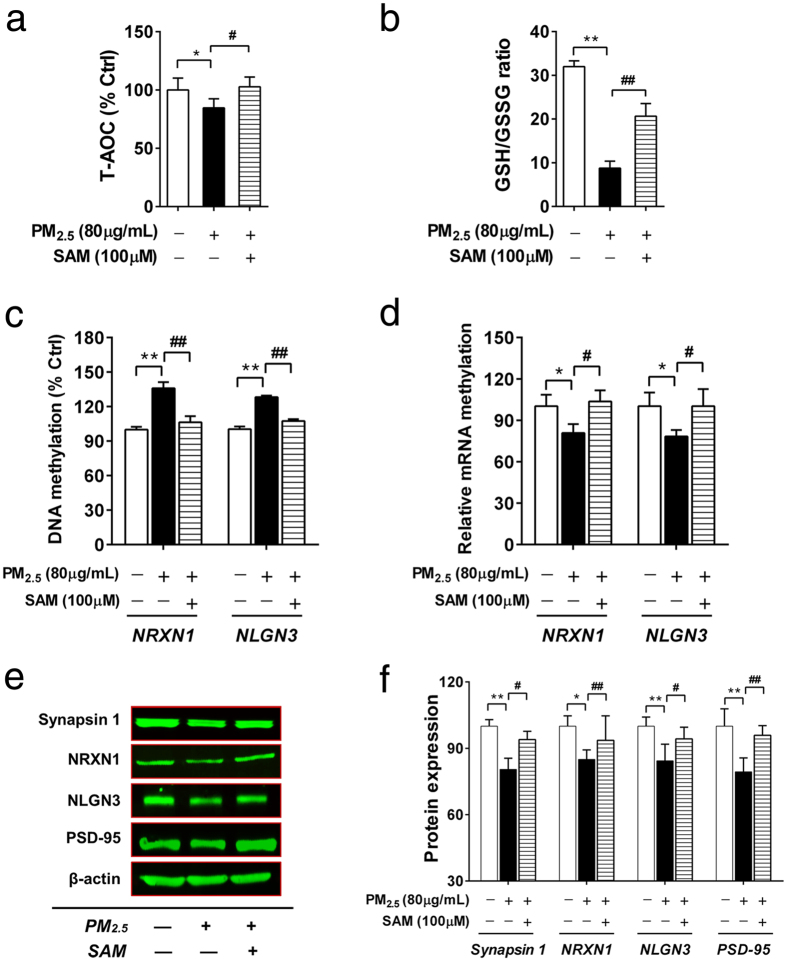
The blocking effects of the methylation supporting agent (SH-SY5Y cells; 72 h treatment). The effects of SAM on PM_2.5_-induced changes in T-AOC ((**a**), n = 6) and GSH/GSSG ((**b**), n = 4). The effects of SAM on PM_2.5_-induced changes in DNA methylation (**c**) and mRNA expression (**d**) of NRXN1 and NLGN3 (n = 3). (**e,f**) The effects of SAM on PM_2.5_-induced changes in protein expression of synapsin 1, NRXN1, NLGN3 and PSD-95. (**e**) The representative images of western blot. (**f**) The quantitation results of protein expression (n = 3). *P < 0.05, **P < 0.01, effects *versus* control; ^#^*P* < 0.05, ^##^*P* < 0.01, effects *versus* PM_2.5_-treated group (by student’s *t* test).

To ultimately confirm the involvement of redox/methylation mechanisms in PM_2.5_-induced abnormal changes, we further investigated the effects of a methylation supporting agent. We found that SAM significantly prevented PM_2.5_-induced decrease in T-AOC and the disturbance in redox imbalance (GSH/GSSG) (Fig. 7a,b), and reversed the global DNA hypomethylation (Supplementary material 1: Fig. S7). As shown in Fig. 7c, SAM significantly inhibited the increase in DNA methylation of NRXN1 and NLGN3 induced by PM_2.5_. The decrease in mRNA expression of NRXN1 and NLGN3 induced by PM_2.5_ were also reversed by SAM (Fig. 7d). SAM further increased the protein expression of synapsin 1, NRXN1, NLGN3 and PSD-95 during PM_2.5_ exposure (Fig. 7e,f). Accordingly, the supplement of SAM reversed H_2_O_2_-induced abnormal changes in DNA methylation and mRNA expression of NRXN1 and NLGN3 (Supplementary material 1: Fig. S8). Finally, the association of promoter DNA hypromethylation of synaptic related genes induced by PM_2.5_ and the decreases in their gene expression was confirmed by the inhibiting effects of DNA methyltransferase inhibitor 5-aza-2′-deoxycytidine (DAC). DAC significantly prevented PM_2.5_-induced increases in DNA methylation, and the decreases in mRNA and protein expression of synapse related genes (Supplementary material 1: Fig. S9). Taken together, these results demonstrated the involvement of redox/methylation mechanisms in PM_2.5_-induced abnormal DNA methylation patterns and synaptic protein expression.

## Discussion

In the current study, we demonstrate that the redox/methylation mechanisms are involved in PM_2.5_-induced abnormal DNA methylation patterns and synaptic protein expression in human neuronal cells. Abnormal changes in DNA methylation patterns included global DNA hypomethylation and gene-specific DNA hyper- or hypomethylation of autism candidate genes. Our findings possibly provide the underlying mechanisms for the neurodevelopmental dysfunction caused by particulate air pollution.

Neurodevelopmental disorders have been considered to be multifactorial disorders, resulting from a complex combination of genetic, epigenetic and environmental factors^21^. Dysregulation of DNA methylation caused by a genetic or environmental insult can result in cognitive deficit and behavior abnormities^22^. We proposed a mechanism of aberrant methylation capacity based on the metabolic relationship between oxidative stress and methylation cycle. The findings of PM_2.5_-induced disturbance in redox balance and methylation capacity together with the blocking effects of antioxidative reagents, demonstrated that PM_2.5_-induced oxidative stress reduced the intercellular levels of SAM and subsequent global DNA methylation. Our results provide biological explanations for the epidemiological association of significant genome hypomethylation in PM_2.5_-exposed population^8^,^9^.

Global DNA hypomethylation is as prevalent as gene-specific methylation abnormities in etiology of chronic disease states. While these two types of epigenetic abnormalities usually affect different DNA sequences. Global DNA hypomethylation is frequently seen in both highly and moderately repeated DNA sequences^23^. Abnormal gene-specific DNA methylation mainly occurs in CpG site of gene promoters with the consequence of silencing or activating target genes. In our work, we further analyzed the influence of PM_2.5_ on gene-specific DNA methylation and correlative mRNA expression in autism candidate genes. These genes are associated with neuron growth and differentiation, neuron spatial orientation and synapse functions and so on. Thus, multiple abnormalities of gene-specific DNA methylation and mRNA expression were induced by PM_2.5_ and its extracts. Studies about etiology of ASD indicate that no single gene can account for more than 1% of the cases of ASD^21^,^24^. Site-specific DNA methylation differences are widespread in monozygotic-discordant ASD twins^25^. Recent study reveals that CpG sites existing in introns are potentially important for alternate splicing with functional consequences for the protein product^26^. Especially, the isoform-specific expression of SHANK3 with alternative splicing is altered in ASD brain tissue with increased SHANK3 methylation in intragenic CpG islands^27^. Although our data suggested the role of promoter CpG methylation and correlative gene expression in neurotoxicity of air pollution, the changes in methylation levels of gene body regions and their influence on the alternative splicing of coding exons should been the focus in future studies.

The brain processes information by transmitting signals at synapses to form vast networks of communicating cells. NRXN1 and NLGN3 are synaptic adhesion molecules that connect presynaptic and postsynaptic neurons, and perform important functions in synaptic transmission. Previous studies have found that the hypermethylated regions in autistic brain samples are associated with neuron-neuron synaptic transmission^16^, which is similar with our results of DNA hypermethylation in NRXN1 and NLGN3. However they didn’t analyze the effects of functional protein expression. Here, we demonstrated that the hypermethylation of NRXN1 and NLGN3 were correlated with the decreases in their mRNA and protein expression. In addition, synapsin 1 and PSD-95 are synapse-associated proteins that are important to synaptic formation, synaptic transmission and axon outgrowth^28^,^29^. Furthermore, our results of decreased protein expression of synapsin 1 and PSD-95 strongly support abnormal synaptic homeostasis caused by PM_2.5_.

A link between epigenetic regulation and antioxidant/detoxification capacity has been reported in many children with autism^15^,^20^. The results showed above indicate that ambient fine particles are potent oxidants that produce oxidative stress and impair the methylation capacity. Furthermore, we found that treatment with the antioxidative reagents NAC, and the supplement with GSH and SAM were successful in preventing PM_2.5_-induced redox/methylation imbalance, DNA hypermethylation and the decreases in synaptic protein expression. NAC is the most commonly used antioxidative reagent, and mainly acts by improving the supply of cysteine for GSH synthesis. NAC and GSH support the supply of GSH in the restoration of redox homeostasis, whereas supplementation with SAM directly provides methyl group to mitigate the stress of GSH synthesis and maintain the normal methylation capacity. The blocking effects of NAC, GSH and SAM confirmed the involvement of a redox/methylation mechanism in PM_2.5_-induced abnormal DNA methylation and synapse homeostasis. Data form human studies have shown that supplementation with a combination of betaine, folinic acid and methylcobalamin, the substrates of the methylation cycle, could normalize the the SAM/SAH and GSH/GSSG ratios^30^,^31^. These evidences may implicate a plausible development of antioxidative protection and methylation support to ameliorate the neurodevelopmental impairment associated with environmental causes.

To evaluate the effects of the chemical constituents of PM_2.5_, we prepared the whole particles of PM_2.5_, water-soluble extracts, organic extracts and carbon core component. Overall, the Pw included both metals with high bioavailability and PAHs with low number of rings^32^,^33^. Po mainly comprised PAHs with high number of rings. Pc was mainly composed of metals with low bioavailability^32^,^33^. The biological effects generally followed a rank order of PM_2.5_ ≥ Pwo > Po > Pw > Pc. Although the contents of metals in Pc are almostly equal to that in Pw, the potential biological effects of Pw may be larger than that of Pc as a result of a greater biological activity of the soluble metals^32^,^33^. However, Pc induced larger cell toxicity than Pw, which may be explained by the mechanical damage resulting from the sedimentation of the particles onto the cell surfaces^34^. The results indicated that more quantities of chemical components (metals and PAHs) in PM_2.5_ induced more extensive and serious effects, and that decreasing the contents of the metals and PAHs could decrease the neurotoxicity of PM_2.5_.

Overall, the present findings have several implications for our understanding the mechanisms for PM_2.5_-induced neurodevelopment disorders. In addition, two additional points should be considered in the interpretation of these results. First, although our findings were performed in neuron cells, the influence of PM_2.5_ on DNA methylation during the neurodevelopmental phase may be more extensive and severe in the context of the global epigenetic reconfiguration occurring during mammalian brain development. Second, the study was performed in cell model and didn’t investigate other possible contributing mechanisms, such as the dysregulated neuroimmune responses. Emerging studies suggest that aberrant immune response possibly change the epigenetic signatures in the brain and induce aberrant epigenetic regulation of microglia^35^. The malfunctioning of the epigenetic machinery in microglia cells, the immune cell in central nervous system, can cause autistic-like behavior^35^. Early postnatal exposure to ultrafine particulate matter has been related to increased neuroinflammatory response and glial activation^36^. Overall, these evidences suggest directions for the future *in vivo* or human study to address the interactions between environmental insults, DNA methylation and neurodevelopment impairment by targeting redox imbalance, immune system activation or other possible mechanisms.

To our knowledge, this is the first report to reveal that redox/methylation mediated abnormal DNA methylation is one of the regulators of PM_2.5_-induced neurodevelopment impairment and synaptic dysfunctions. Our findings may provide a novel epigenetic mechanism for the neurodevelopment toxicity of particulate air pollution, and implicate a plausible development of antioxidative protection and methylation support to ameliorate the neurodevelopmental disabilities associated with environmental causes.

## Methods

### PM_2.5_ collection

The PM_2.5_ sampling site is located in the vicinity of Chongqing South Road in Shanghai, China. Chongqing South Road is a two-lane, two-way vehicle road with a high traffic density. The PM_2.5_ samples were mainly traffic-derived particles and they were collected from 26th Nov. to 21st Dec. 2014 with large-volume PM_2.5_ samplers (Intelligent 2031, Qingdao Laoying Inc., China).

### Preparation of PM_2.5_ and its extracts

The ultrasonic extraction technique was used to obtain the PM_2.5_ suspensions. All of the PM_2.5_ suspensions were divided into two equal parts. Half of the suspension was freeze-dried under vacuum to obtain the PM_2.5_ (the whole particle of PM_2.5_). The other half of the suspensions was used for the preparation of the water-soluble extracts (Pw), the organic extracts (Po) and the carbon core component (Pc) in turn. The protocol for preparation of PM_2.5_ and its extracts is shown in Supplementary Fig. S10 (Supplementary material 1). The prepared samples were weighed and stored at −20 °C. Then we calculated the proportions of every type of extracts. In the current work, the proportions of Pw, Po, and Pc were approximately 50%, 15% and 35%, respectively.

### Chemical analysis of PM_2.5_ and its extracts

The chemical components of PM_2.5_ and its extracts were analyzed by inductively coupled plasma-mass spectrometry (ELAN DRC II, PerkinElmer, America) and inductively coupled plasma atomic emission spectrometry (SPS-8000, Leeman, USA) for 23 types of metals, and gas chromatography-mass spectrometer (Agilent 7890/5975C, Agilent Technologies, USA) for 16 types of PAHs. The list of the chemical components is presented in Supplementary Table S1 (Supplementary material 2).

### Cell culture

We chose the SH-SY5Y human neuroblastoma cell line as the *in vitro* cell model. The SH-SY5Y cells show natural promoter methylation as in the normal brain, and the average percent of the promoter methylation in SH-SY5Y cells is comparable to that in the human cerebral cortex^37^. The SH-SY5Y cells was purchased from Shanghai Institute for Biological Sciences, Chinese Academy of Sciences. The cells were cultured in Modified Eagle’s medium/F12 supplemented with 10% fetal calf serum and 2 mM GlutaMAX^TM^ (Gibco). The SH-SY5Y cells were 100% positive for neuronal markers (microtubule-associated protein 2 and β-tubulin) (Supplementary material 1: Fig. S11).

### Cell treatment

In the present study, the stocking solutions of PM_2.5_, Pw and Pc were prepared with sterilized ddH_2_O in 4 mg/mL, and the stocking solution of Po was prepared with dimethyl sulfoxide (DMSO) in 20 mg/mL. Thus, we chose ddH_2_O and DMSO as the negative controls. As we aimed to reveal the redox/methylation regulatory mechanism, 10 μM of H_2_O_2_ was selected as the positive control according to the results of cytotoxicity and the induction of reactive oxygen species (Supplementary material 1: Fig. S12). For cell viability assay, the cells were treated for 72 h with positive control, negative control and 0, 2.5, 5, 10, 20, 40, 80, 160 and 320 μg/mL PM_2.5_. The concentrations of Pw, Po, Pc and Pwo (the mixture of Pw and Po) were calculated by multiplying PM_2.5_ concentration by their individual proportions (Pw, 50%; Po, 15%; Pc, 35%). The redox/methylation mechanism was evaluated by using antioxidants NAC (5 mM), GSH (10 mM), SAM (100 μM) and DAC (10 μM). All inhibitors were co-applied with 80 μg/mL PM_2.5_ for 72 h.

### Cell viability assay

The cell viability was detected by CCK-8 assay with some modifications. Briefly, the culture plate after incubation with CCK-8 was then centrifuged at 300 g for 3 min, and the reaction mixture in each well was transferred to another 96-well plate to eliminate the impossible impact of the particles on absorbance measurement. The maximum absorbance in 570 nm was measured using the microplate reader (Thermo Scientific Multiskan GO).

### Oxidative stress measurements

Intracellular reactive oxygen species generation was determined using dichloro-dihydro-fluorescein diacetate (DCFH-DA) method as described before. T-AOC was detected by commercially kit with ABTS method (Beyotime Biotechnology, China). The contents of intercellular MDA were detected by commercially kit with thibabituric acid method (Beyotime Biotechnology, China). The concentration of GSH and GSSG were analyzed by commercially kit based on the DTNB method (Beyotime Biotechnology, China). Simultaneously, the protein concentrations were determined using a BCA kit to normalize the results. All procedures were carried out according to the manufacturers’ instructions.

### Determination of intracellular SAM and SAH

An enzyme-coupled continuous spectrophotometric assay was used to determine the levels of intracellular SAM and SAH as previously described^38^,^39^. After treatments, the cells were collected and mixed with ice-cold buffer to prepare the cell lysates. Three freeze-thaw cycles were used to disrupt cell membranes and release the cellular contents. The samples were then centrifuged at 16,000 g for 5 min at 4 °C. The supernatants were removed and assayed for SAM and SAH immediately. The total protein concentrations in the supernatants were determined simultaneously to normalize the results.

### Global 5mC analysis

Immunofluorescence was performed as previously reported^40^. In brief, the genomic 5mC was stained with a monoclonal mouse anti-human 5mC antibody (1: 100, Epigentek) overnight at 4 °C, and subsequently with a Cy3-conjugated anti-mouse IgG (1: 100) for 2 h at room temperature. The nuclei were stained with Hoechst 33258. The stained cells were photographed using a fluorescence microscope (Olympus Corporation, Japan). Captured images were analyzed by measuring the average fluorescence density using Image J program (NIH software). In addition, we quantified the contents of global 5mC by MethyIfIash^TM^ Methylated DNA Quantification Kit (Colorimetric, Epigentek) following the manufacturer’s instructions.

### mRNA expression by qRT-PCR

Total RNA was isolated from the treated cells using the Trizol Reagents (Life Technology). The total RNA in each sample was reverse-transcribed into cDNA with PrimeScript RT reagent Kit (Takara BioInc., China). The levels of the transcripts of the target genes were determined using quantitative PCR (qPCR) with SYBR green reagent (Takara BioInc., China) on a Light Cycler PCR system (Roche). GAPDH was used as an internal control for sample normalization. The 2^−ΔΔCt^ method was used to calculate the relative amount of the target mRNA. The primers for mRNA expression are provided in Supplementary Table S5 (Supplementary material 2).

### MeDIP analysis

The 5mC levels in the promoter regions of autism candidate genes were determined using MeDIP according to the protocol of Methylamp^TM^ Methylated DNA Capture Kit (Epigentek). Briefly, genomic DNA was sheared to generate fragments of approximately 100–600 bp by sonication. Each sample of the sonicated DNA was immunoprecipitated with the human monoclonal 5mC antibody or with normal IgG. DNA from the antibody-bound fractions was purified with Proteinase K in DNA isolation buffer. The purified immunoprecipitated DNA and input DNA were subsequently co-amplified with qPCR and the percent enrichment was calculated by the formula: 100 × 2^(adjusted input Ct−IP Ct)^ as previous described^41^. The primers used for the determination of the 5mC levels in the promoter regions of the target genes are provided in Supplementary Table S6 (Supplementary material 2).

### DNA methylation analysis by bisulfite-pyrosequencing assay

The promoter DNA methylation of synapse related genes was confirmed by pyrosequencing. The genomic DNA was extracted from freshly frozen cells using a commercial DNA Extraction kit with a Spin Column (QIAGEN). The unmethylated cytosine residues in genomic DNA were converted to uracil using bisulfite treatment with the EpiTect Fast Bisulfite Conversion Kit (QIAGEN) according to the manufacturer’s protocol. The bisulfite-treated DNA was then used as a template for the subsequent PCR using a Pyromark PCR kit (QIAGEN). Pyrosequencing was performed on 10 μL of the PCR products. The biotinylated strand was captured on streptavidin-coated beads and incubated with the sequencing primers using a PyroMark Vacuum Prep Workstation (QIAGEN). The pyrosequencing was performed using Pyromark Gold Q90 reagents (QIAGEN). The CpG sites in promoters were analyzed by pyrosequencing using the PyroMark Q24 system (QIAGEN). The primers for the PCR amplification and pyrosequencing are listed in Supplementary Table S7 (Supplementary material 2).

### Western blot

The protein expression of synapse related genes was determined by western blot as described in our previous study^42^. Briefly, the proteins were separated by electrophoresis on an 8% or 6% sodium dodecyl sulfate polyacrylamide gel and transferred onto polyvinylidene fluoride membranes. The membranes were incubated with anti-human synapsin 1 (1: 6,000, CST), NRXN1 (1: 1,000, Abcam), NLGN3 (1: 1,000, Abcam), SHANK3 (1: 200, Santa Cruz), PSD-95 (1: 2,000, CST), and β-actin (1: 5,000, CST) antibody overnight at 4 °C. Then, the strips were incubated with anti-rabbit or anti-mouse or anti-goat IRDye 800CW IgG (1: 15,000, LI-COR) for 1 h at room temperature. The immunoreactivity signals were directly detected using an Odyssey SA Infrared Imaging System (LI-COR Biosciences, USA). The respective intensities were determined using Quantity One software.

### Statistical analysis

All data are presented as the means ± standard deviation. Statistical significance was determined by Student’s *t* test for two group comparisons or one-way ANOVA with the LSD *post hoc* test for multiple group comparisons. All statistical analyses were performed using SPSS 19.0 software. The graphs were produced using GraphPad Prism 6.0 software and HemI software. The level of statistical significance was set at 0.05 (two-tailed).

## Additional Information

**How to cite this article**: Wei, H. *et al*. Redox/methylation mediated abnormal DNA methylation as regulators of ambient fine particulate matter-induced neurodevelopment related impairment in human neuronal cells. *Sci. Rep.* **6**, 33402; doi: 10.1038/srep33402 (2016).

## Supplementary Material

Supplementary Figures

Supplementary Tables
