# Supplementary material for: Redox/methylation mediated abnormal DNA methylation as regulators of ambient fine particulate matter-induced neurodevelopment related impairment in human neuronal cells
Source: Sci Rep. 2016 Sep 14;6:33402. doi: 10.1038/srep33402 (PMC5022064; doi:10.1038/srep33402)
Supplement: Supplementary Figures [file srep33402-s1.pdf]

Supplementary figures

Redox/methylation mediated abnormal DNA methylation as regulators of ambient fine particulate matter-induced neurodevelopment related impairment in human neuronal cells

Hongying Wei[1], Fan Liang[1], Ge Meng[1], Zhiqing Nie[2], Ren Zhou[1], Wei Cheng[1], Xiaomeng Wu[1], Yan Feng[1,]*, Yan Wang[1,3,]*

[1] Shanghai Jiao Tong University School of Public Health; Hongqiao International Institute of Medicine, Shanghai Tongren Hospital, Shanghai 200025, China.

[2] Shanghai Jiao Tong University School of Environmental Science and Engineering, Shanghai, 200240, China.

[3] Shanghai Ninth People's Hospital, Shanghai Jiao Tong University School of Medicine, Shanghai, 200011, China.

*** Corresponding author**

Yan Wang, Ph.D (Email: wangyan@shsmu.edu.cn)

Yan Feng, Ph.D (Email: fy_575@sjtu.edu.cn)

Shanghai Jiao Tong University School of Public Health; Hongqiao International Institute of Medicine, Shanghai Tongren Hospital

No. 227 Chongqing South Road, Shanghai 200025, China

Tel & Fax: 86-21-63846590-776710

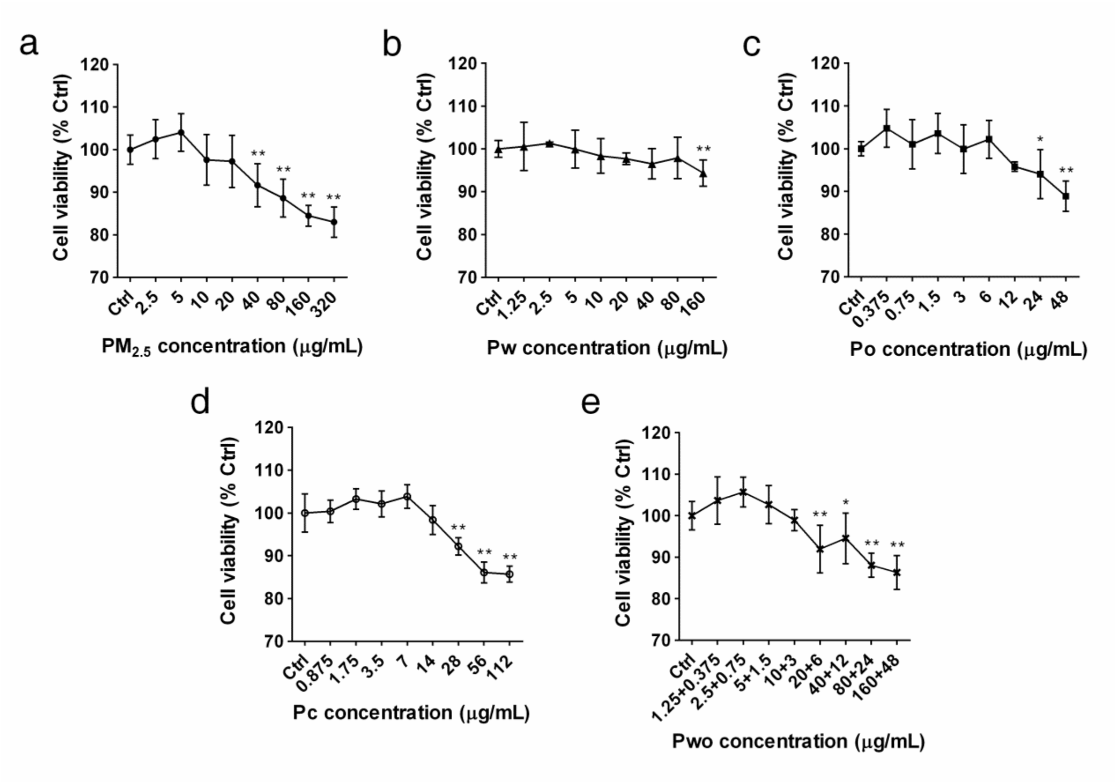

Figure S1

The cell viability in SH-SY5Y cells exposed to $PM_{2.5}$ (a), Pw (b), Po (c), Pc (d) and Pwo (e) with different concentrations for 72 h (n=6). **P*<0.05, ***P*<0.01, effects *versus* control (by one-way ANOVA with LSD *post hoc* test).

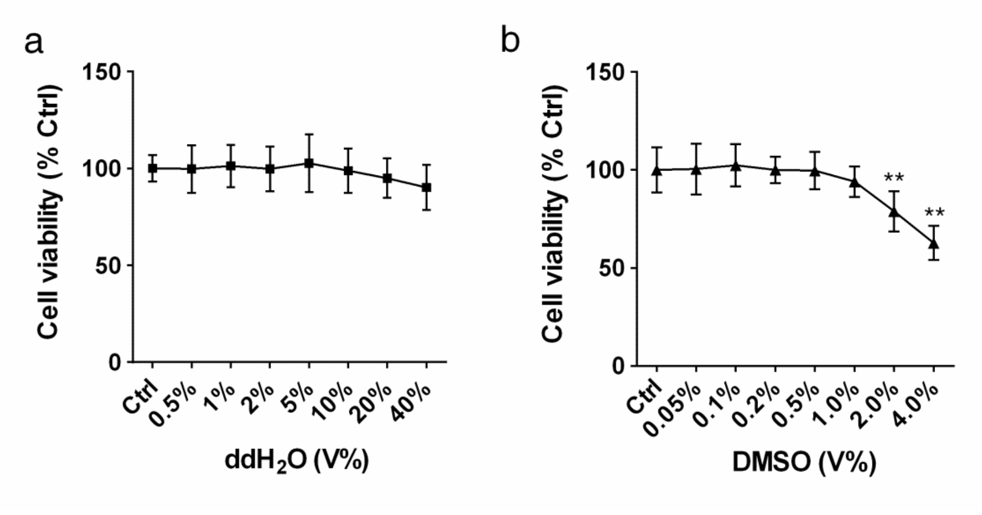

Figure S2

The cell viability in SH-SY5Y cells exposed to ddH_2O (a) and DMSO (b) with different concentrations for 72 h (n=6). **P*<0.05, ***P*<0.01, effects *versus* control (by one-way ANOVA with LSD *post hoc* test).

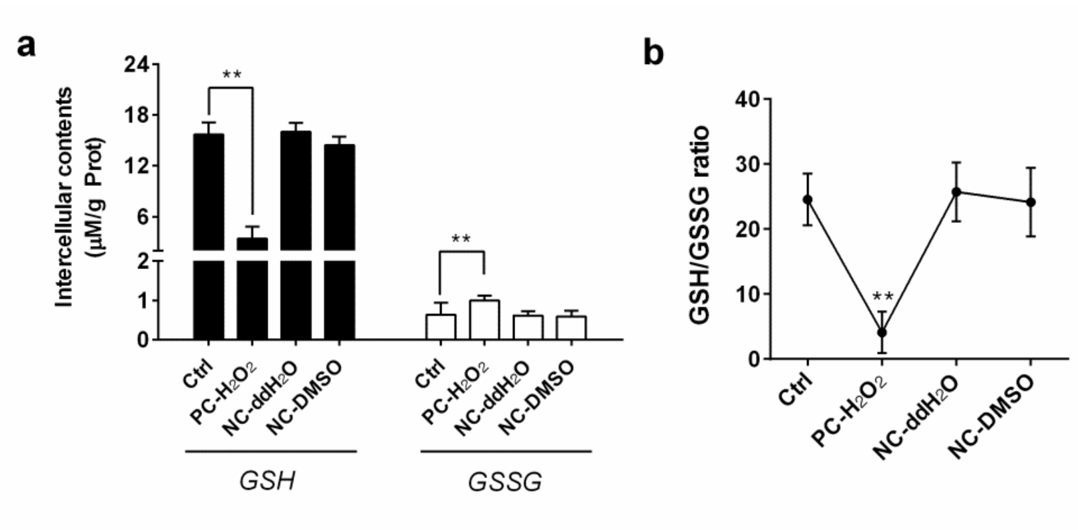

Figure S3

The intracellular GSH (a, n=4), GSSG (a, n=4) and GSH/GSSG (b, n=4) in SH-SY5Y cells exposed to H_2O_2 (10μM), ddH_2O (2.0%) and DMSO (0.1%) for 72 h. PC: positive control. NC: negative control. **P*<0.05, ***P*<0.01, effects *versus* control (by student's *t* test).

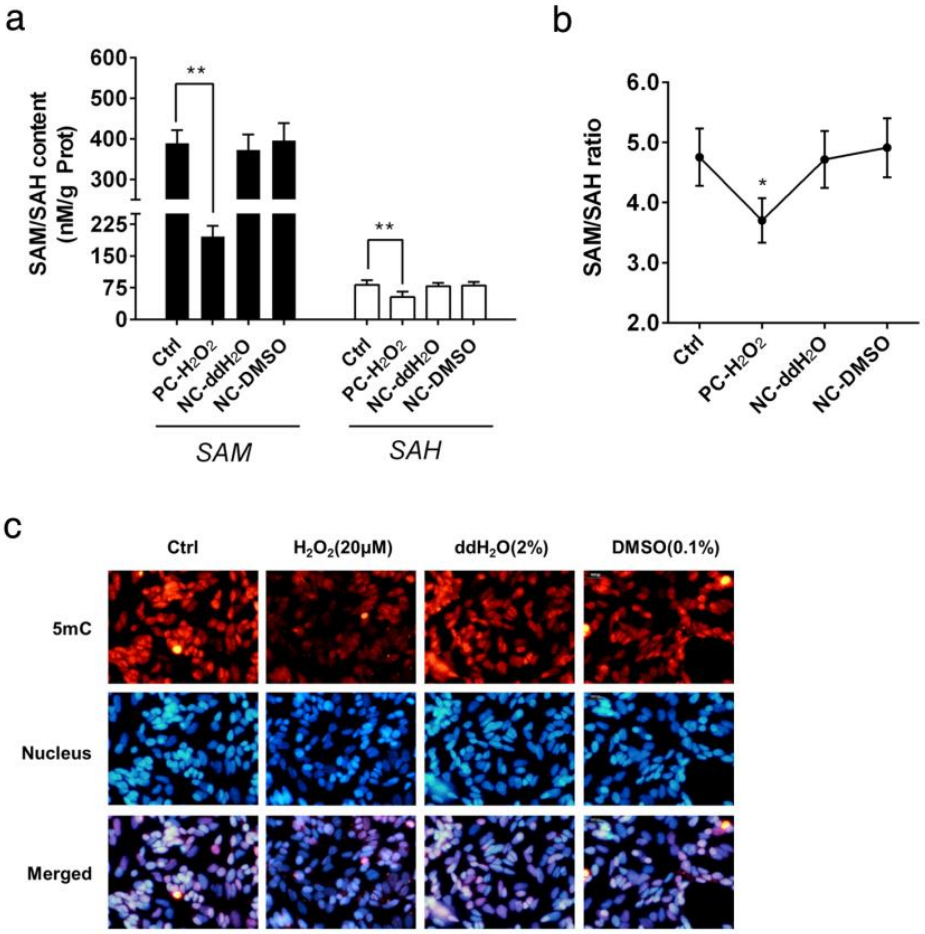

Figure S4

The intracellular SAM (a, n=3), SAH (a, n=3) and SAM/SAH (b, n=3). (c) The representative images of 5mC immunofluorescence (400×). Red: Cy3-5mC. Blue: Hoechst 33258-cell nuclei. The SH-SY5Y cells were exposed to H_2O_2 (10μM), H_2O (2.0%) and DMSO (0.1%) for 72 h. PC: positive control. NC: negative control. *P<0.05, **P<0.01, effects *versus* control (by student's *t* test).

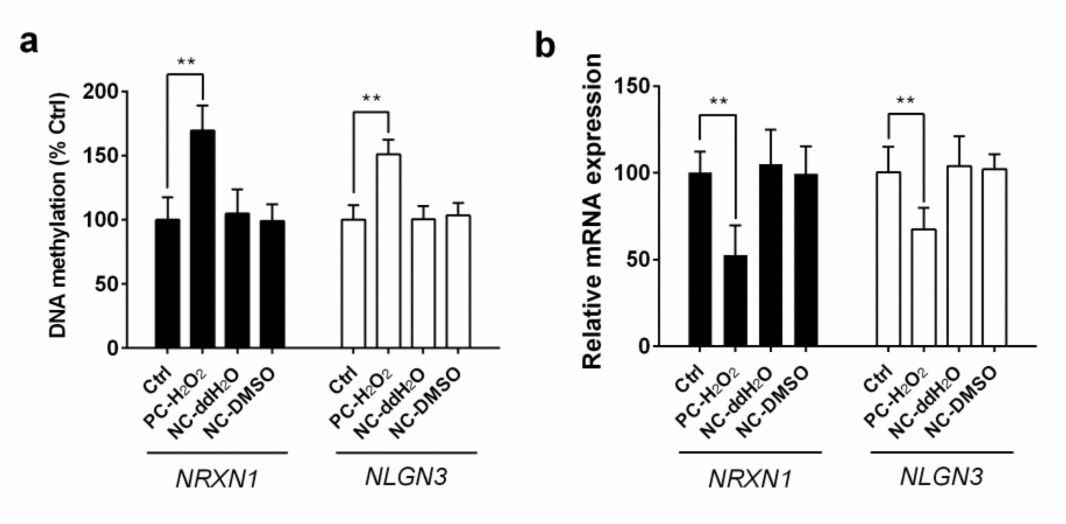

Figure S5

DNA methylation (a) in promoter regions of NRXN1 and NLGN3 (n=3). Relative mRNA expression (b) of NRXN1 and NLGN3 (n=3). The SH-SY5Y cells were exposed to H_2O_2 (10μM), ddH_2O (2.0%) and DMSO (0.1%) for 72 h. PC: positive control. NC: negative control. **P*<0.05, ***P*<0.01, effects *versus* control (by student's *t* test).

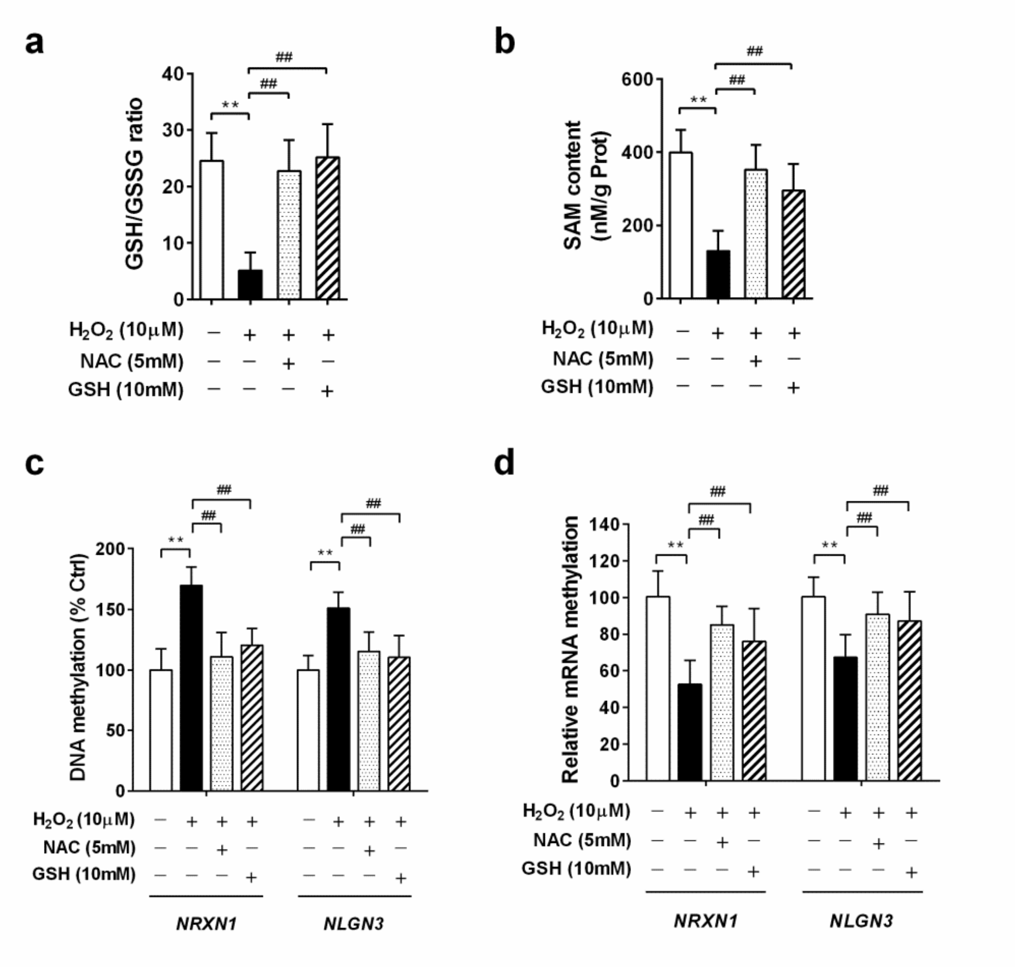

Figure S6

The effects of NAC and GSH on H_2O_2-induced changes in GSH/GSSG (a, n=4) and SAM content (b, n=3). The effects of NAC and GSH on H_2O_2-induced changes in DNA methylation (c) and mRNA expression (d) of NRXN1 and NLGN3 (n=3). **P*<0.05, ***P*<0.01, effects *versus* control; #*P*<0.05, ##*P*<0.01, effects *versus* H_2O_2-treated group (by student's *t* test).

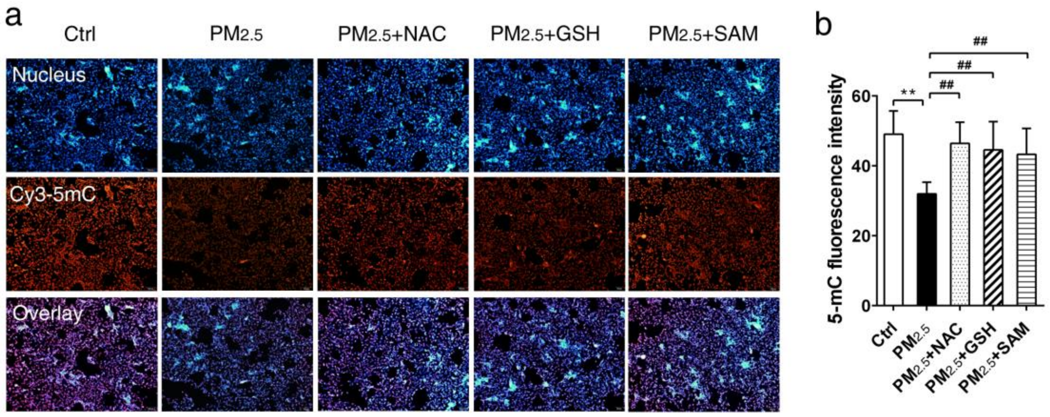

Figure S7

Effects of antioxidative reagents and methylation supporting agent on $PM_{2.5}$-induced global DNA hypomethylation. (a) 5-mC immunofluorescence (200×) in SH-SY5Y cells treated for 72 h with 80 μg/mL $PM_{2.5}$ alone or with NAC (5 mM), GSH (10 mM) or SAM (100μM). Blue: Hoechst 33258-stained cell nuclei. Red: Cy3-5mC. (b) The quantitative results of 5-mC fluorescence intensity (n=3). *P<0.05, **P<0.01, effects *versus* control; $^{\#}P$<0.05, $^{\#\#}P$<0.01, effects *versus* $PM_{2.5}$ group (by student's *t* test).

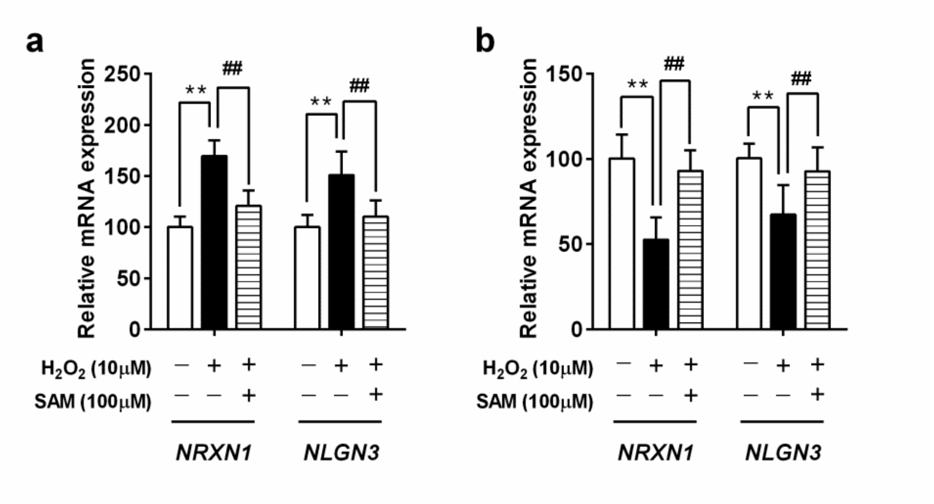

Figure S8

The effects of SAM on H_2O_2-induced changes in DNA methylation (a) and mRNA expression (b) of NRXN1 and NLGN3 (n=3). *P<0.05, **P<0.01, effects *versus* control; $^{\#}P$<0.05, $^{\#\#}P$<0.01, effects *versus* H_2O_2-treated group (by student's *t* test).

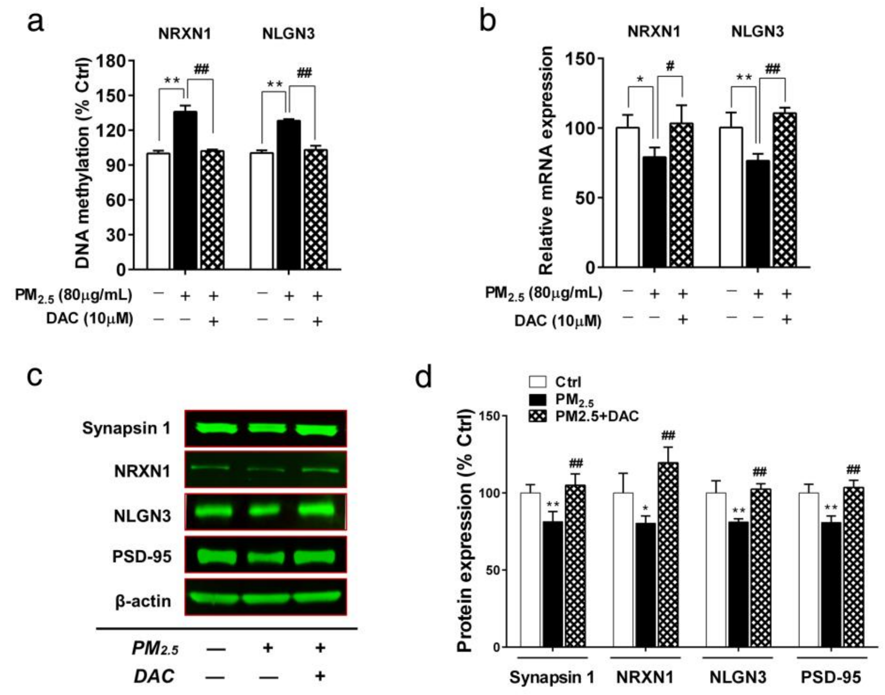

Figure S9

The blocking effects of the DNA methyltransferase inhibitor. The effects of DAC (10 μM) on promoter DNA methylation (a) and mRNA expression (b) of NRXN1 and NLGN3 induced by $PM_{2.5}$ (n=3). (c) and (d) The effects of DAC (10 μM) on protein expression of synapsin 1, NRXN1, NLGN3 and PSD-95 induced by $PM_{2.5}$. (c) The representative image of western blot. (d) The quantitative results of the protein expression (n=3). *P<0.05, **P<0.01, effects *versus* control; $^{\#}P$<0.05, $^{\#\#}P$<0.01, effects *versus* $PM_{2.5}$ group (by student's *t* test).

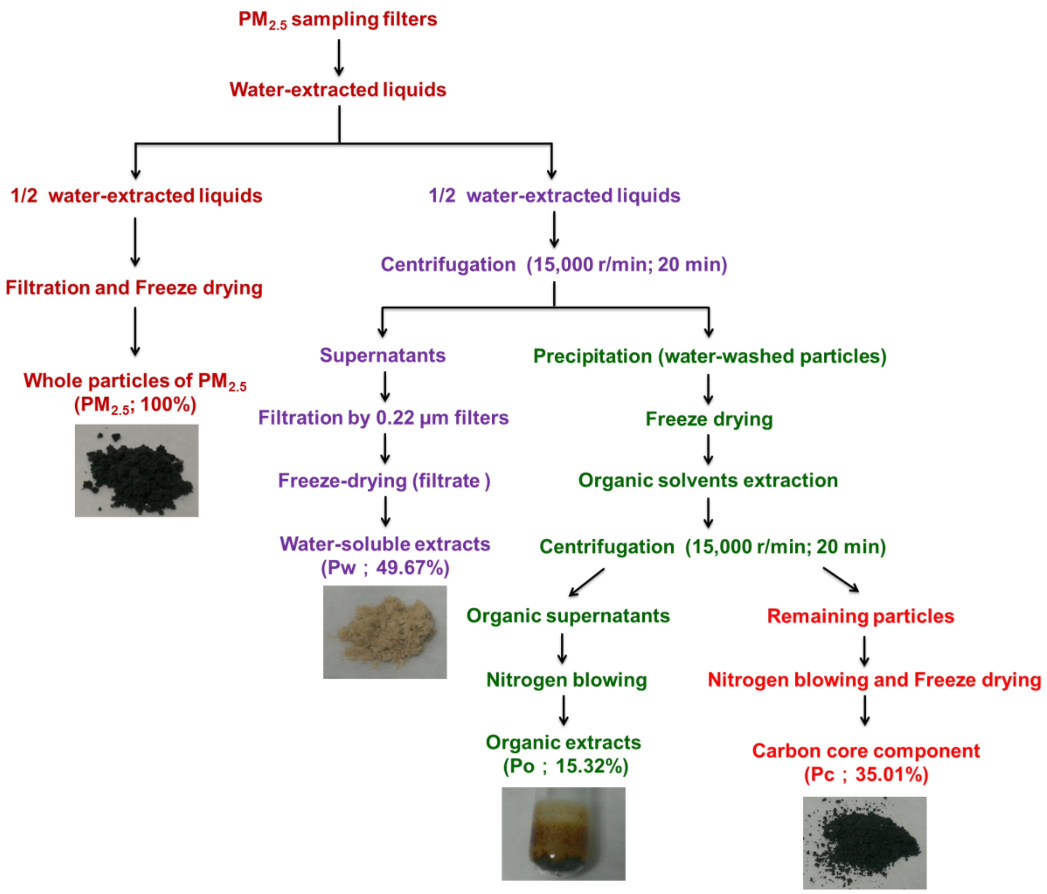

Figure S10

The protocol for the preparation of $PM_{2.5}$ and its extracts. $PM_{2.5}$: the whole particle of $PM_{2.5}$; Pw: the water-soluble extracts of $PM_{2.5}$; Po: the organic extracts of $PM_{2.5}$; Pc: the carbon core component of $PM_{2.5}$.

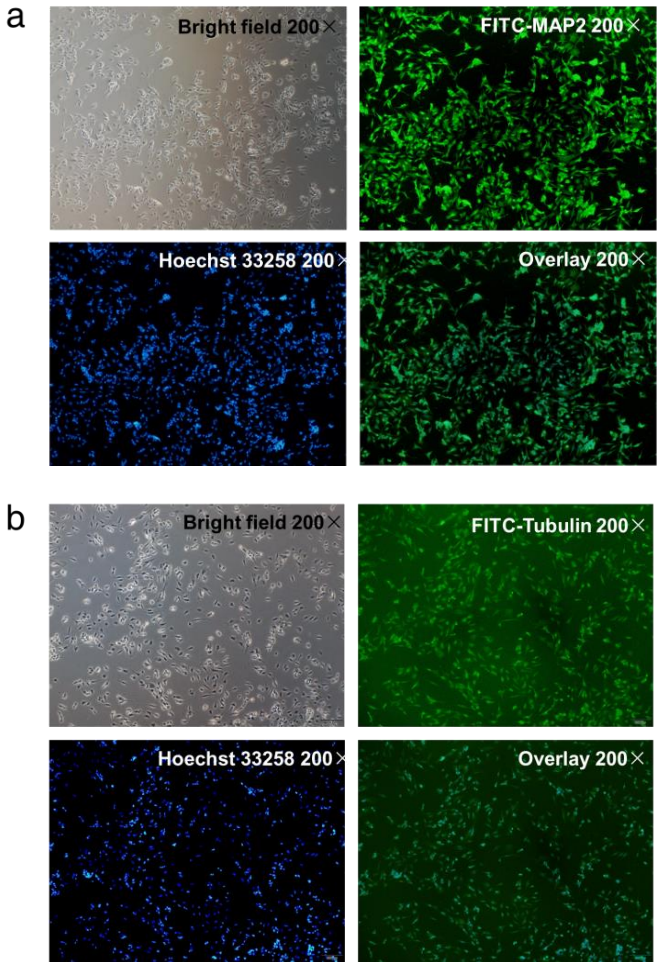

Figure S11

SH-SY5Y cells are positive for the neuron-specific markers. (a) Microtubule-associated protein 2 (MAP2) immunostaning. (b) β-Tubulin immunostaning.

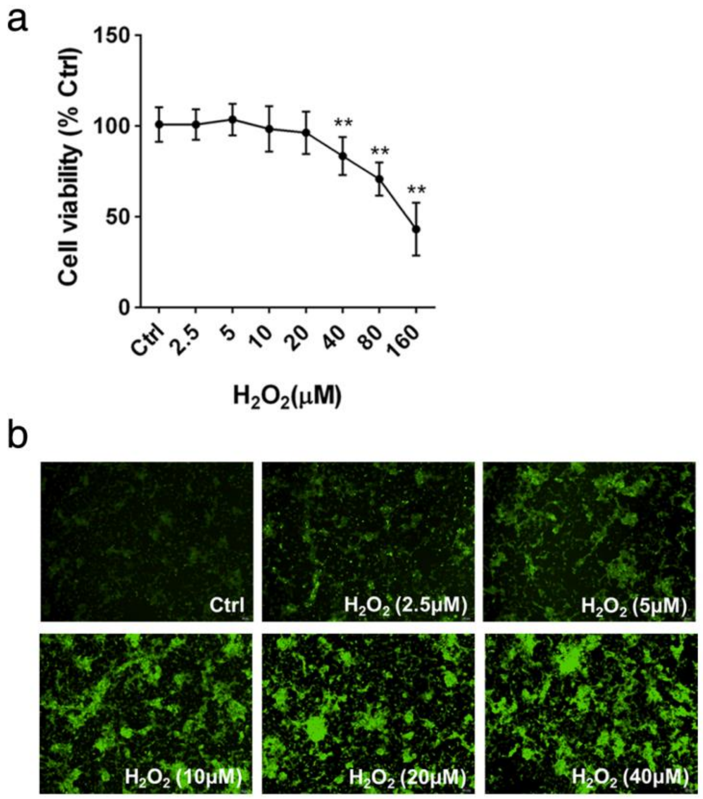

Figure S12

The cell viability (a) and intercellular ROS (b) in SH-SY5Y cells exposed to H_2O_2 with different concentrations for 72 h (n=6). *P<0.05, **P<0.01, effects *versus* control (by one-way ANOVA with LSD *post hoc* test). Note: 10μM of H_2O_2 induced substantial generation of ROS with no obvious cytotoxicity.
