# Supplementary material for: Redox/methylation mediated abnormal DNA methylation as regulators of ambient fine particulate matter-induced neurodevelopment related impairment in human neuronal cells
Source: Sci Rep. 2016 Sep 14;6:33402. doi: 10.1038/srep33402 (PMC5022064; doi:10.1038/srep33402)
Supplement: Supplementary Tables [file srep33402-s2.pdf]

Supplementary tables

Redox/methylation mediated abnormal DNA methylation as regulators of ambient fine particulate matter-induced neurodevelopment related impairment in human neuronal cells

Hongying Wei[1], Fan Liang[1], Ge Meng[1], Zhiqing Nie[2], Ren Zhou[1], Wei Cheng[1], Xiaomeng Wu[1], Yan Feng[1,]*, Yan Wang[1,3,]*

[1] Shanghai Jiao Tong University School of Public Health; Hongqiao International Institute of Medicine, Shanghai Tongren Hospital, Shanghai 200025, China.

[2] Shanghai Jiao Tong University School of Environmental Science and Engineering, Shanghai, 200240, China.

[3] Shanghai Ninth People's Hospital, Shanghai Jiao Tong University School of Medicine, Shanghai, 200011, China.

*** Corresponding author**

Yan Wang, Ph.D (Email: wangyan@shsmu.edu.cn)

Yan Feng, Ph.D (Email: fy_575@sjtu.edu.cn)

Shanghai Jiao Tong University School of Public Health; Hongqiao International Institute of Medicine, Shanghai Tongren Hospital

No. 227 Chongqing South Road, Shanghai 200025, China

Tel & Fax: 86-21-63846590-776710

Table S1 The contents of various chemical components of $PM_{2.5}$ and its extracts

	$PM_{2.5}$	Sum of three extracts	the different extracts		
			Pw	Po	Pc
Crustal metals					
Al, μg/g	26774.14	20657.11	337.88	2437.22	17882.02
Ca, μg/g	44390.97	42016.01	26110.50	1246.60	14658.92
Na, μg/g	28683.80	22864.59	7473.75	4162.85	11227.99
K, μg/g	14711.84	14284.33	9768.75	507.09	4008.49
Mg, μg/g	5990.65	5519.06	2286.75	252.37	2979.94
Sr, μg/g	184.58	184.18	112.33	6.34	65.52
Ba, μg/g	377.92	332.71	44.43	18.63	269.66
Transition metals					
Fe, μg/g	15269.47	13153.97	237.00	944.07	11972.90
Zn, μg/g	2827.10	2700.86	1712.63	154.22	834.02
Cu, μg/g	227.71	204.50	53.87	14.33	136.30
Ti, μg/g	972.66	806.32	10.64	53.85	741.83
Ni, μg/g	62.38	58.17	19.66	4.20	34.30
Mo, μg/g	32.52	34.92	24.78	1.78	8.36
Cd, μg/g	14.14	14.02	7.43	0.80	5.79
V, μg/g	75.28	72.25	29.64	4.15	38.47
Cr, μg/g	119.06	106.93	11.63	8.74	86.57
Mn, μg/g	729.47	686.26	282.94	53.89	349.43
Other metals					
As, μg/g	72.06	60.33	33.06	5.07	22.20
Se, μg/g	51.28	55.05	40.68	2.93	11.45
Sb, μg/g	54.60	49.05	22.77	3.74	22.55
Pb, μg/g	435.60	445.98	17.02	36.05	392.91
Rb, μg/g	54.95	55.46	35.68	1.95	17.83
Cs, μg/g	5.84	5.94	3.56	0.25	2.13
PAHs					
NAP, μg/g	1.39	1.39	1.04	0.21	0.13
ANY, μg/g	0.00	0.00	0.00	0.00	0.00
ANA, μg/g	0.00	0.00	0.00	0.00	0.00
FLU, μg/g	23.61	36.32	26.04	5.19	5.09

Continued Table S1

PHE, μg/g	5.56	6.65	0.00	6.65	0.00
ANT, μg/g	0.00	0.00	0.00	0.00	0.00
FLT, μg/g	0.00	0.00	0.00	0.00	0.00
PYR, μg/g	12.50	12.70	9.38	1.65	1.67
BaA, μg/g	23.61	21.61	12.50	6.70	2.41
CHR, μg/g	6.94	7.94	3.13	3.95	0.87
BbF, μg/g	19.44	16.44	4.17	11.01	1.27
BkF, μg/g	12.50	10.29	3.13	6.36	0.80
BaP, μg/g	12.50	14.16	2.08	11.67	0.40
IPY, μg/g	40.28	30.28	3.13	26.22	0.94
DBA, μg/g	1.39	1.73	0.00	1.66	0.07
BPE, μg/g	16.67	12.80	1.04	11.35	0.40

Notes: $PM_{2.5}$: the whole particle of $PM_{2.5}$; Pw: the water-soluble extracts of $PM_{2.5}$; Po: the organic extracts of $PM_{2.5}$; Pc: the carbon core component of $PM_{2.5}$.

Table S2 The brief introduction of 16 target genes (reference: NCBI GeneBank)

Genes	Gene ID	Name	Functions in neurodevelopment
MeCP2	4204	Methyl CpG binding protein 2	Neurodevelopment. Neuron growth, differentiation and maturation. (Rett syndrome, Autism)
GRIN1	2902	N-methyl D-aspartate receptor 1	Glutamate receptor. Synapse plasticity. Memory and learning.
RELN	5649	Reelin	Cell positioning and neuronal migration. (Schizophrenia, Autism, Bipolar disorder)
EN2	2020	Engrailed homeobox 2	Neuron migration and spatial orientation.
BDNF	627	Brain derived neurotrophic factor	Neuron growth, differentiation and maturation. Neuronal survival. Stress response.
AUTS2	26053	Autism susceptibility candidate 2	Neurodevelopment. Neuron migration and spatial orientation. (Autism, Intellectual disability)
NRXN1	9378	Neurexin 1	Cell adhesion molecules and receptors. Synapse formation and synaptic plasticity
NLGN3	54413	Neuroligin 3	Neuronal cell surface proteins. Site-specific ligands for neurexins. (Asperger syndrome)
SHANK3	85358	SH3 and multiple ankyrin repeat domains 3	Multidomain scaffold proteins. Synapse formation and dendritic spine maturation. (Autism)
SLC6A4	6532	Solute carrier family 6 member 4	Neurotransmitter symporter family. Transports the neurotransmitter serotonin.
GABRB3	2562	Gamma-aminobutyric acid A receptor, beta 3	Receptor for inhibitory neurotransmitter. Neocortical excitation/inhibition balance. (Autism)
GAD1	2571	Glutamate decarboxylase 1	Production of gamma-aminobutyric acid. Neocortical excitation/inhibition balance. (Seizures)
UBE3A	7337	Ubiquitin protein ligase E3A	Ubiquitin protein degradation system. Motor and intellectual development. (Angelman Syndrome)
GABRA5	2558	Gamma-aminobutyric acid A receptor, alpha 5	Receptor for inhibitory neurotransmitter. Neocortical excitation/inhibition balance.
GABRG3	2567	Gamma-aminobutyric acid A receptor, gamma 3	Receptor for inhibitory neurotransmitter. Neocortical excitation/inhibition balance.
AFF2	2334	AF4/FMR2 family, member 2	folate-sensitive fragile X E locus on chromosome X. (Fragile X E syndrome, Mental retardation)

Table S3 The mRNA expression of 16 target genes

Gene name	Ctrl	$PM_{2.5}$	Pw	Po	Pc	Pwo
MeCP2	100.08±4.92	130.86±6.98**	124.84±1.50**	116.71±9.31*	94.25±9.36	126.83±9.77**
GRIN1	100.21±8.05	116.02±5.42*	96.71±12.43	135.35±1.09**	99.08±11.60	97.09±3.7
RELN	100.72±15.11	155.03±11.43**	116.84±14.86	131.56±14.65*	114.64±3.29	131.56±14.65**
EN2	101.23±18.96	96.98±10.43	104.15±11.16	79.64±7.97*	97.86±6.65	96.17±2.34
BDNF	100.21±7.96	83.34±2.32*	75.93±8.68**	80.73±7.86**	107.68±9.05	73.63±7.42**
AUTS2	100.31±9.80	79.02±6.96**	85.29±2.77*	81.88±8.19*	92.56±9.68	77.39±1.54**
NRXN1	100.27±9.12	84.11±2.19**	81.85±3.83**	80.48±6.61**	87.22±6.53*	83.36±3.2**
NLGN3	100.32±9.72	79.41±3.08**	89.81±9.30	75.48±7.07**	79.83±4.56**	72.42±3.38**
SHANK3	100.38±10.47	111.20±8.91	110.76±4.33	77.29±8.00**	97.57±9.27	79.08±7.95**
SLC6A4	100.22±8.20	59.46±0.63**	74.13±4.12**	67.68±0.97**	80.17±7.63*	70.88±10.41**
GABRB3	100.07±4.77	70.42±2.83**	84.58±8.59*	103.94±7.38	84.77±8.39*	88.88±7.28
GAD1	100.74±14.63	83.04±11.22	102.29±7.63	105.97±2.58	98.04±5.22	113.23±15.27
UBE3A	100.59±13.49	96.21±4.20	102.77±16.52	116.37±7.73	90.5±12.31	104.8±4.62
GABRA5	100.02±2.44	106.04±5.51	107.08±11.53	98.74±13.03	102.9±13.43	91.51±9.8
GABRG3	100.1±5.51	113.44±12.03	97.30±8.73	105.26±17.35	101.44±16.99	95.16±12.28
AFF2	100.08±4.95	99.16±28.01	101.70±9.48	96.00±10.31	96.09±16.88	102.49±13.8

Notes: *P<0.05, **P<0.01, effects *versus* control (by one-way ANOVA with LSD *post hoc* test).

Table S4 The 5-mC levels in the promoter regions of 11 target genes

Gene name	Ctrl	$PM_{2.5}$	Pw	Po	Pc	Pwo
MeCP2	100.00±8.39	65.52±13.20**	73.71±14.85*	64.61±13.02**	84.09±16.94	63.11±12.71**
AUST2	100.00±5.87	75.26±6.79*	82.82±13.14	79.01±4.99*	79.53±12.62*	73.34±14.70**
RELN1	100.00±10.05	85.44±13.26	93.01±17.17	89.95±8.03	91.91±9.86	83.34±17.19
BDNF	100.00±7.33	138.81±10.18**	139.47±15.72**	121.21±9.04*	112.97±8.29	142.41±10.44**
NRXN1	100.00±9.46	147.73±11.81**	146.63±10.77**	154.86±12.38**	126.64±10.12*	161.55±19.65**
NLGN3	100.00±9.98	149.25±17.45**	134.00±13.38*	137.12±11.46**	130.24±13.00*	143.13±19.92**
SHANK3	100.00±13.86	130.99±13.08*	125.28±12.51*	135.92±17.21**	114.84±11.46	141.28±14.10**
SLC6A4	100.00±9.98	155.97±15.57**	153.79±14.07**	167.10±16.68**	142.41±14.22**	155.73±12.28**
GABRB3	100.00±19.94	138.51±15.47**	148.45±8.73**	145.48±12.52**	122.26±13.94	150.83±15.58**
GRIN1	100.00±9.98	106.49±10.03	98.55±13.37	95.18±10.00	100.03±9.99	97.95±15.02
EN-2	100.00±9.22	99.58±14.08	94.33±9.42	96.47±9.63	110.96±11.08	105.81±10.56

Notes: The data in this table are the relative results of methylation rate compared to control. The methylation rate in control or treated groups represents the percent of methylated cytosines of CpG sites detected. The percent was calculated form the data of fluorescence intensity by the formula: $100 \times 2^{(\text{adjusted input Ct} - \text{IP Ct})}$. *$P$<0.05, **$P$<0.01, effects *versus* control (by one-way ANOVA with LSD *post hoc* test).

Table S5 The primers of 16 target genes and GAPDH used in qRT-PCR

Genes	Primers (5'to 3')	Bases
MeCP2	Forward primer: GTGGAGTTGATTGCGTACTTCG	22
	Reverse primer: CCCTCTCCCAGTTACCGTGAA	21
BDNF	Forward primer: TAACGGCGGCAGACAAAAAGA	21
	Reverse primer: TGCACTTGGTCTCGTAGAAGTAT	23
EN2	Forward primer: CCGGCGTGGGTCTACTGTA	19
	Reverse primer: CCTCTTTGTTCGGGTTCTTCTT	22
SHANK3	Forward primer: GTCCTGCTCTTCCGTGGAG	19
	Reverse primer: TGGGTCTTGATAACCTCTGCAA	22
RELN	Forward primer: CAACCCCACCTACTACGTTCC	21
	Reverse primer: TCACCAGCAAGCCGTCAAAAA	21
NLGN3	Forward primer: ACAGTGGTGCTAAACCCGTC	20
	Reverse primer: ATTGCCATAACTGGCGAGGAT	21
AUTS2	Forward primer: TCAGATCGAGAAAATGACCGC	21
	Reverse primer: GCTGAACTATCACTGAGCCTTT	22
NRXN1	Forward primer: TAAGTGGCCTCCTAATGACCG	21
	Reverse primer: TCGCACCAATACGGCTTCTTT	21
GRIN1	Forward primer: ACGCCATCCTAGTTAGCCATC	21
	Reverse primer: GCACGGGTATGCGGTAGAAG	20
UBE3A	Forward primer: CTCAGCTTACCTTGAGAACTCG	22
	Reverse primer: TTCTAGCGCCTTTCTTGTTCAT	22
SLC6A4	Forward primer: ACGGAGTTCTACAGAAGGTTGT	22
	Reverse primer: ATAGAGTGCCGTGTGTCATCT	21
GAD1	Forward primer: GCTTCCGGCTAAGAACGGT	19
	Reverse primer: TTGCGGACATAGTTGAGGAGT	21
GAD2	Forward primer: TTTTGGTCTTTCGGGTCGGAA	21
	Reverse primer: TTCTCGGCGTCTCCGTAGAG	20
GABRB3	Forward primer: GATAAAAGGCTCGCCTATTCTGG	23
	Reverse primer: GATCATGCGGTTTTTCACTGTC	22
GABRA5	Forward primer: CATCGCTCACAACATGACCAC	21
	Reverse primer: CCATCGGGAAGTCCTCAAGC	20
GABRG3	Forward primer: AACCGACCGTAATTGACGTTG	21
	Reverse primer: CTGTCCAGGTCTGAGCAAAAA	21
GAPDH	Forward primer: GGAGCGAGATCCCTCCAAAAT	21
	Reverse primer: GGCTGTTGTCATACTTCTCATGG	23

Table S6 The primers for analysis of 5mC levels in promoter regions of 11 target genes

Genes	Chromosome location	Primer sequence (5'-3')	5' site	3' site	Primer length	Product length
RELN	chr7:103471784-103989516	F: CTCTCCTTCCCTCACGCATC	-542	-523	20	126
		R: CTTCTGCGAGGGACGTCAAA	-417	-436	20	
EN2	chr7:155458129-155464831	F: GCAAATGGAGCCGCATACTG	-1271	-1252	20	72
		R: GGACAGCGACCCTCATGAAA	-1200	-1219	20	
MeCP2	chrX:154021573-154097755	F: GCCCACTAAACCAGTCCCTC	-303	-284	20	85
		R: ACCCCTCCAGCTGTTGATTG	-219	-238	20	
SHANK3	chr22:50674415-50733298	F: AAGTCCGGAGCAAACTCCC	-884	-865	20	91
		R: GGCCCTCCGAATTAACCCTT	-794	-813	20	
BDNF	chr11:27654893-27720779	F: CCCACCCACTTTCCCATTCA	-583	-564	20	135
		R: CGGAGGTAATACTCGCACCC	-449	-468	20	
NLGN3	chrX:71144862-71170293	F: TTCTCACGTGACCCACCTTG	-1135	-1116	20	100
		R: TCCAAATGTGACTCTGGGGC	-1036	-1055	20	
AUTS2	chr7:69599333-70793068	F: CGCTGGAGATTTCTTTCTGCT	-249	-229	21	75
		R: GAGGTCACCCTTCCTCAAAA	-175	-194	20	
NRXN1	chr2:49921739-51032200	F: CCAAGGCAGAGCCACAGTAA	-369	-350	20	75
		R: AAGACTCACACAGGCACTGG	-295	-314	20	
GRIN1	chr9:137139481-137168755	F: CTGACTGTGGGTGACGACTC	-363	-344	20	125
		R: GGAAACGTTAGAGGGGTCCG	-239	-258	20	
SLC6A4	chr17:30194319-30236002	F: CTAGGTGGCACCAGAATCCC	-1080	-1061	20	132
		R: TGGAGGAACTGACCCCTGAA	-949	-968	20	
GABRB3	chr15:26543546-26773074	F: AGTCCGGCGGGCAGA	-513	-499	15	83
		R: CGAAGTTGGCCCCGCA	-431	-446	16	

Table S7 The primers for PCR amplification and pyrosequencing of NRXN1 and NLGN3

Genes	Strand	Sequence
NRXN1	F	TTTGGTATAGTTTAGGTAAGTTATGAAGAT
	R	ACCCTTAAAAAAAAACTACAACTTTTC
	S	GTGGGGTAAGGGATA
NLGN3	F	GTTTGGTTTTAGGAGGAAGAGAGAT
	R	TCCATTATAATAAACCAATTAACTCTCC
	S	TGGTAGTTATTTAGGATGAT
